# Supplementary figures and images for: GABAergic neurons are susceptible to BAX-dependent apoptosis following isoflurane exposure in the neonatal period
Source: PLoS One. 2021 Jan 12;16(1):e0238799. doi: 10.1371/journal.pone.0238799 (PMC7802958; doi:10.1371/journal.pone.0238799)

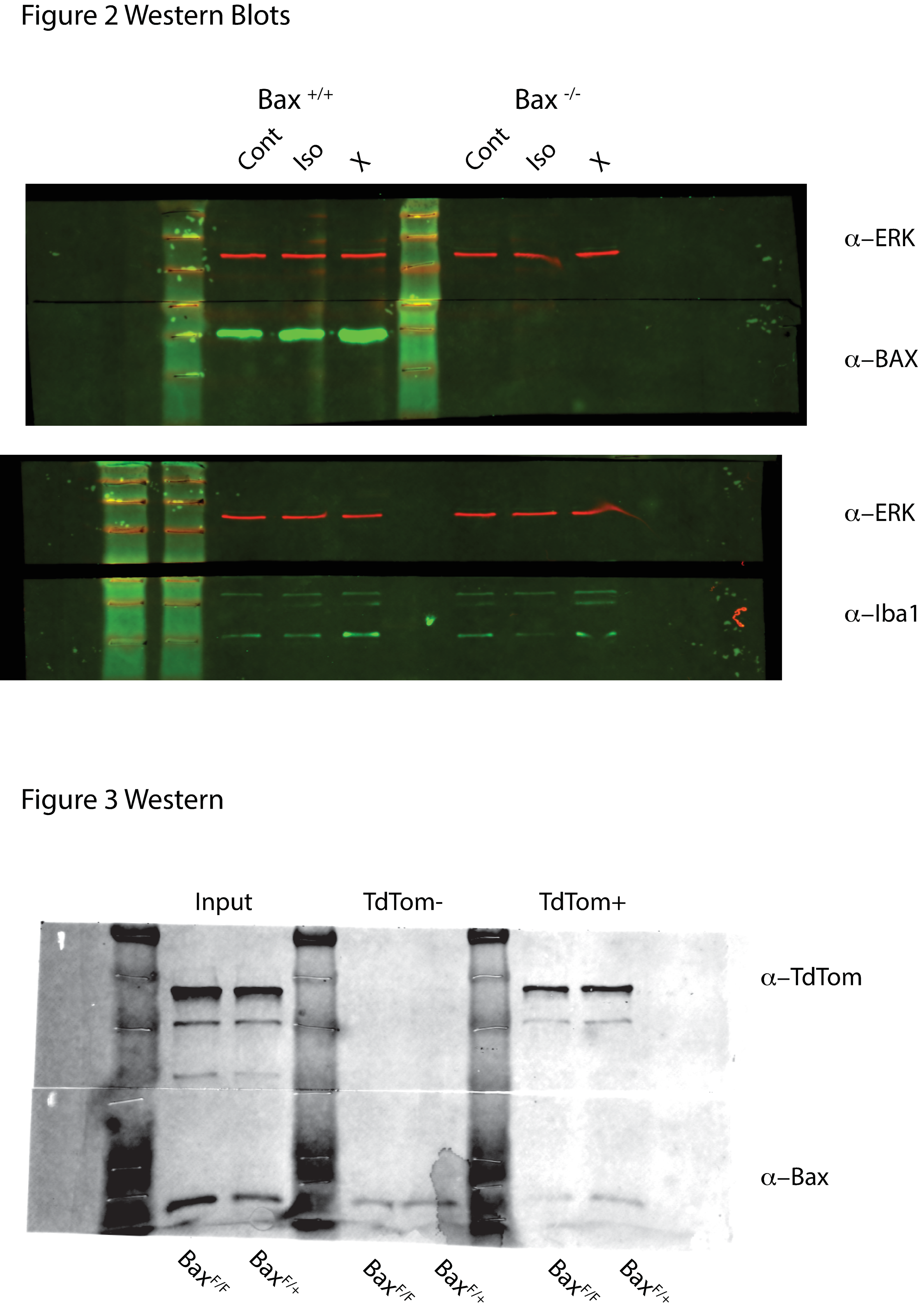

Supplement: S1 Raw images — (TIF) [file pone.0238799.s001.tif]
